# Instrumented Three-Dimensional Gait Analysis in an Adult With KIF1A-Associated Neurological Disorder: A Case Report

**DOI:** 10.7759/cureus.112579

**Published:** 2026-07-13

**Authors:** Ana Rita Pessoa, Diogo Portugal, Fábio Pinho, Inês Camarinha, Jorge Jacinto

**Affiliations:** 1 Physical and Rehabilitation Medicine Service, Unidade Local de Saúde de São José, Lisboa, PRT; 2 Adult Rehabilitation Service, Centro de Medicina e de Reabilitação de Alcoitão, Lisboa, PRT

**Keywords:** botulinum toxin, gait analysis, hereditary spastic paraplegia, kand, kif1a, kinematics

## Abstract

*KIF1A*-associated neurological disorder (KAND) encompasses recessive and dominant variants with wide clinical variability. Several de novo variants in the *KIF1A* gene have been reported to cause a complicated form of hereditary spastic paraplegia (HSP), frequently accompanied by peripheral neuropathy, cerebellar ataxia, and cognitive impairment. Instrumented three-dimensional (3D) gait analysis is underutilized in this population, and comparative data between KAND and pure HSP remain limited. We performed a comprehensive instrumented 3D gait analysis in a 19-year-old woman with KAND caused by a heterozygous de novo *KIF1A* variant c.773C>T (p.Thr258Met) in the motor domain (exon 8), presenting with progressive spastic paraparesis. Assessment included spatiotemporal parameters; 3D kinematics of the hip, knee, and ankle; kinetic analysis of ground reaction forces; joint moments and powers; baropodometry; surface dynamic telemetric electromyography (EMG) of the lower limb muscles bilaterally; and fine-wire EMG of the left flexor digitorum longus and flexor hallucis longus.

Walking speed, stride length, and cadence were within normal limits for age and sex. Kinematic analysis revealed bilateral deficits in hip and knee extension during stance, hip internal rotation and adduction during swing, reduced knee flexion in swing, and bilateral ankle dorsiflexion deficit during swing. Clinically, maintained flexion of all toes bilaterally was observed and filmed. Ankle push-off power was markedly reduced bilaterally (32.5%-42.5% of normative values). EMG demonstrated out-of-phase activation of the adductor longus, gracilis, semimembranosus, and biceps femoris, and continuous co-contraction throughout the gait cycle of the extensor digitorum longus, flexor digitorum longus, and flexor hallucis longus. Brief high-amplitude bursts compatible with possible myoclonic activity were identified in the right gastrocnemius and soleus during swing phase.

This case illustrates a KAND phenotype in which gait speed is preserved, but kinematic, kinetic, and EMG profiles are substantially abnormal, a pattern that differs from classical HSP, where speed reduction is typically a hallmark finding. The combination of spastic, ataxic, and peripheral neuropathy components produces a distinct and complex gait signature. Quantitative gait analysis may provide clinically useful information to guide targeted interventions, including botulinum toxin injections, orthotic management, and physiotherapy, in this heterogeneous population.

## Introduction

*KIF1A*-associated neurological disorder (KAND) is a clinically heterogeneous, progressive neurodegenerative group of inherited or de novo conditions caused by pathogenic variants in the *KIF1A* gene, which encodes a kinesin-3 motor protein responsible for the anterograde axonal transport of synaptic vesicles, dense core vesicles, and organelles in neurons. Variants in the *KIF1A* gene -- primarily affecting the motor domain -- disrupt the transport of synaptic vesicles containing synaptophysin and synaptotagmin, leading to a broad spectrum of phenotypes, most notably spastic paraplegia and peripheral neuropathy [1].

This rare condition may also manifest as intellectual disability, cerebellar atrophy, optic nerve atrophy, and microcephaly [2-5]. Seizures are among the most common associated features [2-4]. Cerebellar dysfunction has been reported in a significant proportion of patients, and dysautonomic symptoms, including temperature instability and urinary retention, may also occur [6]. In severe disease, gastrointestinal dysfunction may lead to parenteral nutrition [6]. Disease severity and onset are strongly influenced by the location and type of *KIF1A* variant: missense mutations in the motor domain are generally associated with earlier onset and more severe phenotypes [2,7,8]. De novo variants in the *KIF1A* gene are frequently misdiagnosed, often as cerebral palsy or other more prevalent spastic disorders [1,2,4].

Reported presentations include early developmental delay and gait abnormalities that emerge at the onset of independent walking, including equinus gait, unsteady gait, and later a progressive spastic paraplegia pattern [9,10] -- which may ultimately lead to loss of ambulation [3,9,11,12]. Mobility deterioration is attributed to progressive increase of spasticity, muscle weakness, and secondary contractures, sometimes associated with peripheral neuropathy and cerebellar ataxia [9,10].

Available instrumented gait analysis data in KAND patients suggest a spectrum ranging from mild spastic gait -- characterized by reduced range of motion at the hip, knee, and ankle; increased knee flexion at initial contact; and delayed peak knee flexion -- to severe crouch gait, depending on the specific variant and disease severity [10]. In affected children, it has demonstrated reduced gait speed, decreased stride length, increased double-support time, and abnormal trunk and pelvic movements [10].

These findings partially overlap with the broader spectrum of hereditary spastic paraplegia (HSP), where instrumented gait analysis has revealed similar spatiotemporal and kinematic abnormalities [13-17]. However, the coexistence of peripheral neuropathy and cerebellar ataxia in KAND may further exacerbate gait instability and differentiate it from pure HSP or spastic diplegic cerebral palsy [9,10].

Despite the recognized impact of gait impairment in KAND, instrumented gait analysis data in this population remain extremely scarce. Only one published study -- Van Beusichem et al. -- has characterized gait using three-dimensional (3D) analysis, specifically in patients with de novo *KIF1A* variants, describing four pediatric cases aged 10-18 years [10]. We found no published adult case report incorporating a comprehensive instrumented gait analysis -- kinematics, kinetics, surface and fine-wire (FW) electromyography (EMG), and baropodometry -- in an adult KAND patient. This case report aims to contribute to filling this gap by describing the detailed gait profile of a 19-year-old woman with a confirmed heterozygous de novo *KIF1A* variant (p.Thr258Met) and contextualizing the findings within the existing HSP and KAND literature.

## Case presentation

A 19-year-old active woman with KAND, caused by a heterozygous de novo* KIF1A* variant c.773C>T (p.Thr258Met) in the motor domain (exon 8 of NM_001244008.1), was referred for instrumented gait analysis. The variant was initially identified at age 9 by whole-exome sequencing with next-generation sequencing, with alignment to GRCh37 and variant annotation via Ensembl VEP, GEMINI, and Alamut pipelines, and confirmed by Sanger sequencing. It is absent from gnomAD and in-house control databases. At the time of the initial genetic report (2016), the variant was classified as of uncertain clinical significance. Repeat genetic sequencing in 2018 supported reclassification toward a pathogenic interpretation, in keeping with emerging functional evidence -- most notably the demonstration of a dominant-negative effect on *KIF1A* motor activity reported by Cheon et al. -- and pathogenicity has since become the dominant classification across the literature and major variant databases [18]. The diagnosis of KAND was established following clinical correlation with the patient's progressive spastic paraparesis phenotype and review of the subsequent literature on motor-domain *KIF1A* variants.

The patient presented with progressive spastic paraparesis, with perceived reduced strength in the left lower limb. She was ambulatory without aids at the time of assessment (February 2026) and was enrolled in a customized physiotherapy program targeting lower limb strength, neurocoordination, spasticity management, and gait efficiency. Clinical examination noted bilateral callosities over the dorsal surface of the toes and a symmetrical postural pattern with claw-toe deformity (with extension of the metatarsophalangeal joints and flexion of the interphalangeal joints of all toes). Morning plantar pain, bilaterally, was clinically interpreted as myofascial tension of the quadratus plantae.

Instrumented gait analysis protocol

3D gait analysis was performed in March 2026 at the Gait Analysis Laboratory, Centro de Medicina de Reabilitação de Alcoitão (CMRA), Alcabideche, Portugal. Motion capture was performed using a Vicon system (Vicon Motion Systems Ltd., Oxford, United Kingdom; 8× T10 and 2× VERO infrared cameras) operating at 120 Hz, with retroreflective markers placed according to the Vicon Plug-in-Gait model. Two Basler piA1000-48gc digital video cameras (Basler AG, Ahrensburg, Germany) provided synchronized lateral and frontal video. Sequences were acquired with the patient walking barefoot, without orthotic devices, walking aids, or external support.

Ground reaction forces were recorded using four AMTI OR6-7-2000 force plates (Advanced Mechanical Technology, Inc., Watertown, Massachusetts, USA) at 1000 Hz. Plantar pressure distribution was assessed with an RsScan® 2 m long baropodometric platform (RSscan International, Paal-Beringen, Belgium). Electromyographic data were collected using the Cometa Mini Wave Infinity wireless system (Cometa Systems S.r.l., Bareggio, Milan, Italy; surface and FW electrodes). FW electrodes were inserted under high-resolution ultrasound guidance (Siemens ACUSON NX3, Siemens Healthineers, Erlangen, Germany; 5.0-10 MHz linear transducer) to confirm placement in the left flexor digitorum longus (FDL) and left flexor hallucis longus (FHL). The following muscles were assessed by EMG bilaterally, unless stated otherwise (Table 1).

**Table 1:** Muscles assessed by surface and fine-wire EMG EMG, electromyography; FW, fine wire.

Anterior compartment/medial thigh	Posterior compartment
Rectus femoris, adductor longus, gracilis	Semimembranosus, biceps femoris
Tibialis anterior, extensor digitorum longus	Gastrocnemius, soleus, flexor digitorum longus (FW — left), flexor hallucis longus (FW — left)

Spatiotemporal parameters (cadence, walking speed, stride and step length, step width, double support time, single support time, and foot-off timing) were computed for both lower limbs. 3D kinematics of the pelvis, hip, knee, and ankle were analyzed across the full gait cycle. Kinetic analysis included ground reaction forces, intersegmental joint moments, and joint powers, with particular attention to ankle push-off power generation in terminal stance.

Results

Spatiotemporal Parameters

Spatiotemporal parameters were within normal limits for age and sex [19], with mean values of six trials (Table 2). Mean walking speed was 1.16 m/s (left) and 1.12 m/s (right). Cadence was 126 steps/min (left) and 123 steps/min (right). Stride length was 1.11 m (left) and 1.10 m (right). A mild asymmetry in stance phase duration was noted, with slightly reduced right stance time (foot off at 57.7% of gait cycle on the right compared with 59.3% on the left), suggesting slightly reduced confidence in right-limb loading.

**Table 2:** Spatiotemporal gait parameters (mean ± SD) SD, standard deviation.

Parameter	Left	Right
Walking speed (m/s)	1.16 ± 0.067	1.12 ± 0.095
Cadence (steps/min)	126 ± 3.78	123 ± 6.99
Stride length (m)	1.11 ± 0.040	1.10 ± 0.046
Step length (m)	0.54 ± 0.016	0.56 ± 0.038
Step width (m)	0.089 ± 0.021	0.080 ± 0.027
Foot off (% gait cycle)	59.3 ± 1.64	57.7 ± 1.46
Double support (s)	0.16 ± 0.032	0.16 ± 0.030
Single support (s)	0.41 ± 0.026	0.40 ± 0.033
Stride time (s)	0.96 ± 0.029	0.98 ± 0.060
Limp index	1.04 ± 0.033	0.98 ± 0.032

Kinematics

Pelvic kinematics revealed rotation of 15º-20º toward the right throughout the gait cycle.

At the hip, extension deficit was observed bilaterally during stance phase (12º deficit on the right, 8º on the left -- considering 20º as normal hip extension) [19]. Swing phase hip flexion was adequate bilaterally, with slightly increased flexion of approximately 35º. A pattern of hip internal rotation and adduction was present during swing phase. 

At the knee, stance phase extension deficit in mid-stance was 5º on the right and 8º on the left -- considering 0º as normal knee extension [19]. During swing phase, reduced peak knee flexion was identified, with a 15º deficit on the right and 5º on the left -- considering 50º as normal knee flexion [19].

At the ankle and foot, initial contact was plantigrade bilaterally, consistent with bilateral pes cavus, confirmed by static and dynamic baropodography findings (simultaneous rearfoot and forefoot contact). Bilateral dorsiflexion deficit of 5º was recorded during swing phase, considering 10º as normal maximal ankle dorsiflexion [19]. On visual and video observation, a bilateral claw-toe pattern (as described before) was present throughout the gait cycle.

Kinetics

Ground reaction forces showed peak weight transfer at 10% of the gait cycle bilaterally, without clinically significant deviations in joint moment profiles. Ankle push-off power was markedly reduced bilaterally, reaching only 42.5% of normative values [19], on the right and 32.5% on the left. This reduction in terminal stance power generation limits anterior propulsion of the center of mass, reduces the ability to flex the hip during swing, and contributes to compensatory strategies in proximal segments and trunk, in order to achieve normal gait speed (efficacy of gait).

Electromyography

Rectus femoris demonstrated a normal activation pattern bilaterally. Adductor longus and gracilis showed out-of-phase activation during swing phase bilaterally, consistent with a spastic pattern contributing to hip adduction and internal rotation in swing. Semimembranosus showed out-of-phase activation at mid-stance and early swing on the left; the right side was within normal limits. Biceps femoris showed out-of-phase activation at mid-stance and early swing on the right; the left was within normal limits. Tibialis anterior demonstrated normal activation bilaterally.

Extensor digitorum longus was active throughout the entire gait cycle bilaterally, indicating compensatory co-contraction contributing to the claw-toe deformity. FW EMG was performed unilaterally on the left FDL and FHL, both of which showed continuous activity throughout the gait cycle -- a pathological activation pattern consistent with toe flexion drive, equinus tendency, and impaired dorsiflexion during swing. Because clinical examination and visual gait analysis demonstrated comparable findings bilaterally, the right-sided interpretation was based on extrapolation rather than direct FW recording, thereby avoiding unnecessary contralateral invasive assessment.

Right gastrocnemius and right soleus each demonstrated two discrete bursts of out-of-phase activation during swing phase. The morphology of these bursts -- brief, high-amplitude discharges during an otherwise electrically quiescent phase -- is suggestive of possible myoclonic activity, though this finding requires further neurophysiological characterization. Left gastrocnemius and soleus were within normal limits (Table 3). 

**Table 3:** EMG findings summary EMG, electromyography; FW: fine wire.

Muscle	Side	Finding	Pattern
Rectus femoris	Bilateral	Normal	—
Adductor longus	Bilateral	Out-of-phase in swing	Spastic/pathological
Gracilis	Bilateral	Out-of-phase in swing	Spastic/pathological
Semimembranosus	Left	Out-of-phase mid-stance/early swing	Spastic/pathological
Semimembranosus	Right	Normal	—
Biceps femoris	Right	Out-of-phase mid-stance/early swing	Spastic/pathological
Biceps femoris	Left	Normal	—
Tibialis anterior	Bilateral	Normal	—
Extensor digitorum longus	Bilateral	Continuous throughout gait cycle	Co-contraction
Gastrocnemius	Right	Two bursts during swing	Possible myoclonic
Gastrocnemius	Left	Normal	—
Soleus	Right	Two bursts during swing	Possible myoclonic
Soleus	Left	Normal	—
Flexor digitorum longus (FW)	Left	Continuous throughout gait cycle	Pathological
Flexor hallucis longus (FW)	Left	Continuous throughout gait cycle	Pathological

## Discussion

This case report describes the detailed instrumented gait profile of a 19-year-old woman with KAND caused by a confirmed heterozygous de novo *KIF1A* variant p.Thr258Met, located in the motor domain. The most clinically striking finding is the dissociation between preserved spatiotemporal parameters and substantial deviation from normal in kinematic, kinetic, and EMG profiles -- a pattern that has not been emphasized in prior KAND gait reports, to our knowledge, and one that differs from the typical presentation in pure HSP.

In the existing HSP literature, gait speed reduction is commonly reported as a core feature and a sensitive marker of disease severity and progression [13-16]. Studies using inertial sensors and 3D motion capture in HSP adults have demonstrated that stride length, cadence, and walking speed are among the most reliable indicators of motor pathway dysfunction [17]. In the only published pediatric gait analysis study in *KIF1A*-related spastic paraplegia, Van Beusichem et al. described four children aged 10-18 years, two of whom were unable to walk without assistance and exhibited severe crouch gait with marked contractures [10]. The present case demonstrates that a KAND patient may reach early adulthood with preserved walking speed yet display a complex and clinically significant gait pathology, underscoring the inadequacy of speed-based gait assessments alone to characterize gait dysfunction in this population.

The kinematic profile observed here is consistent with an upper motor neuron pattern, possibly superimposed on concomitant dysfunctions from peripheral and cerebellar contributions. Bilateral hip and knee extension deficits during stance, combined with reduced knee flexion in swing, mirror the spastic pattern described in HSP [14,15]. Pulido-Valdeolivas et al. identified six distinct gait phenotypes in pediatric HSP, with patterns characterized by anterior pelvic tilt, hip flexion at initial contact, and delayed peak knee flexion in swing -- features also present in our case [14]. Similarly, Piccinini et al. demonstrated that EMG co-contraction patterns in HSP closely resemble those observed in spastic diplegia [16].

The ankle kinetics are of particular clinical significance. Push-off power was reduced to 42.5% (right) and 32.5% (left) of normative values [19] -- a substantial bilateral deficit in the terminal stance propulsive mechanism. Although reduced ankle power has been reported in HSP [15,16], and in spastic cerebral palsy [20], the magnitude of the deficit observed here may plausibly reflect a compounded interaction between upper motor neuron dysfunction, peripheral neuropathy involving the distal muscles, and continuous toe-flexor co-contraction mechanically impairing ankle rocker function. However, this remains a pathophysiological hypothesis based on gait findings and requires confirmation through longitudinal assessment and targeted neurophysiological studies. The EMG findings merit particular attention. The continuous activation of FDL and FHL -- confirmed by FW electrodes placed intramuscular under ultrasound guidance -- represents a clear pathological pattern driving the claw-toe deformity and contributing to the deficit of ankle dorsiflexion in swing. The bilateral co-contraction of extensor digitorum longus throughout the gait cycle is probably a compensatory strategy to increase ankle dorsiflexion in swing, but at the same time, it increases the claw deformity during stance by creating an antagonist force pair across the toes. This specific combination of extrinsic toe muscle dysfunction has not been previously characterized by FW EMG in KAND.

The possible myoclonic bursts identified in the right gastrocnemius and soleus during the swing phase represent a potentially novel finding. Episodic muscle discharges during the swing phase -- a period of minimal expected soleus/gastrocnemius activity -- may reflect central excitability abnormalities consistent with the known epileptiform propensity of KAND [2,3]. Alternatively, they may represent spastic clonic activity triggered by rapid unloading of the limb. Neurophysiological confirmation with simultaneous electroencephalogram-EMG polygraphy would be required to characterize these discharges further.

Future cohort studies should determine whether the coexistence of spastic, peripheral neuropathic, and possibly cerebellar gait components in KAND renders the gait signature distinctly complex compared with pure HSP. Wolf et al. demonstrated that instrumented gait analysis can distinguish HSP from cerebral palsy through specific kinematic signatures [21]. We suggest that similarly rigorous analysis may distinguish KAND from both pure HSP and spastic cerebral palsy, particularly when FW EMG and kinetic data are integrated.

From a therapeutic perspective, the gait analysis findings directly inform the rehabilitation plan. Botulinum toxin injection into adductor longus and gracilis bilaterally may be considered by the treating team to reduce hip internal rotation and adduction in swing. Semimembranosus (left) and biceps femoris (right) are candidates for injection to improve knee extension in stance. FDL and FHL bilaterally -- with findings from the left extrapolated to the right based on clinical and video assessment -- are primary targets to address the claw-toe pattern and improve ankle dorsiflexion in swing, ankle coronal alignment in stance, and potentially the pes cavus deformity. Extensor digitorum longus may also benefit from treatment (maybe in a sequential approach and if needed) given its contribution to the claw-toe deformity. These recommendations are consistent with the broader HSP literature supporting botulinum toxin as a component of a multimodal rehabilitation approach [13]. Custom insoles were also recommended to support the objectively quantified cavus deformity. To accommodate potential improvements following treatment, these should only be prescribed four to six weeks after botulinum toxin injection.

This case report has several limitations. As a single case, generalizability is limited, and the findings may not be representative of the full phenotypic spectrum of KAND. FW EMG was restricted to the left lower limb for technical reasons, limiting direct bilateral comparison of deep compartment muscles. Longitudinal data, including post-intervention follow-up, are absent at this moment (but suggested to the treating team who referred the patient), precluding assessment of treatment response, disease progression, and the persistence of the proposed gait signature over time. Standardized clinical rating scales, where applicable, were not systematically and longitudinally applied, limiting the correlation between clinical severity and instrumented gait findings. In addition, neurophysiological data were limited, particularly for characterizing peripheral neuropathy and myoclonus, thereby limiting the interpretation of their contribution to the gait phenotype. To further develop the knowledge on this topic, future studies should combine comprehensive instrumented gait analysis with standardized clinical, longitudinal, post-intervention, and neurophysiological assessments in larger KAND cohorts, with normative comparisons against age-matched HSP populations.

## Conclusions

To our knowledge, this is the first published case report describing a comprehensive instrumented 3D gait analysis -- incorporating kinematics, kinetics, baropodometry, and surface and FW EMG -- in an adult patient with KAND. The key finding is the preservation of spatiotemporal parameters in the context of substantially altered kinematic, kinetic, and EMG profiles, a dissociation that distinguishes this case from typical HSP and highlights the limitations of functional walking assessments alone in KAND.

This gait signature reflects the multisystem nature of KAND, with upper motor neuron, peripheral neuropathic, and possible cerebellar contributions producing a complex and clinically actionable profile. Instrumented gait analysis may be a valuable tool for the clinical management and monitoring of KAND patients, guiding targeted interventions and enabling objective longitudinal monitoring of disease progression and treatment response.
